# A clinical study of efficacy and safety of the Carry Life UF system in continuous ambulatory peritoneal dialysis patients: protocol for a prospective, multicenter, randomized, crossover study

**DOI:** 10.1186/s12882-025-04095-2

**Published:** 2025-04-03

**Authors:** Martin Wilkie, Charlotte de Leon, Ola Carlsson, Jörgen Hegbrant, Olof Heimbürger

**Affiliations:** 1 Sheffield Kidney Institute, Sheffield Teaching Hospitals, Sheffield, UK; 2 Triomed AB, Lund, Sweden; 3 Division of Nephrology, Department of Clinical Sciences, Lund University, Lund, Sweden; 4 Medical Unit Renal Medicine, Karolinska University Hospital, and CLINTEC, Karolinska Institutet, Stockholm, Sweden

**Keywords:** Peritoneal dialysis, Steady concentration peritoneal dialysis, Ultrafiltration, Sodium removal, Glucose, Carry life UF system

## Abstract

**Background:**

Carry Life UF is a novel peritoneal dialysis (PD) technology for improved fluid management using steady concentration PD (SCPD). The Carry Life UF treatment starts with a manual peritoneal fill of 1.36% glucose PD fluid, followed by a 5-hour treatment where small amounts of glucose are continuously added to maintain a stable intraperitoneal glucose concentration. A recent in-center clinical study using the Carry Life UF system demonstrated higher ultrafiltration (UF) rates, more efficient use of glucose (increased UF volume/gram of glucose absorbed), and greater sodium removal with the Carry Life UF treatments compared with a 2.27% glucose continuous ambulatory PD (CAPD) dwell. The aim of this study is to compare efficacy and safety of the Carry Life UF system with a standard CAPD prescription in the home setting.

**Methods:**

A prospective, multicenter, randomized, crossover study of 19 adult subjects at up to 12 sites in Italy, Sweden and the UK will complete the investigation. End-stage kidney disease patients with a CAPD prescription of 2–4 exchanges per day, including at least one 2.27% glucose dwell, will be included. After a Carry Life UF glucose dose determination phase performed in-clinic, subjects will be randomized to start the home treatment part of the study with either the control arm (2.27% glucose CAPD dwell) or the Carry Life UF arm (11 or 15 g/h glucose dose), each for four weeks. The primary endpoint is UF volume comparing the control CAPD 2.27% glucose dwell with the Carry Life UF treatment. Secondary endpoints include adverse event rates, peritoneal sodium removal, glucose UF efficiency, and peak dialysate glucose concentration.

**Discussion:**

This study will evaluate a novel PD technology in the home environment. Challenging aspects include the need to accurately measure UF volumes at home and to support subjects in using a novel technology. The study design considers important parameters for precise UF volume measurements and provides detailed weighing instructions to the study team to ensure consistency between study centers. Research nursing support will be provided for training of subjects and to support endpoint data collection in the subjects’ home. Due to the significant burden associated with the study, subjects will be offered a fair compensation, in accordance with local regulations.

**Trial registration:**

ClinicalTrials.gov Identifier: NCT05874804 Registration date: 18th of April 2023.

**Supplementary Information:**

The online version contains supplementary material available at 10.1186/s12882-025-04095-2.

## Background

Achieving adequate peritoneal ultrafiltration (UF) and sodium removal is a challenge in peritoneal dialysis (PD). Despite volume management being an important component of PD prescription [1, 2], volume overload remains a common problem for PD patients with a prevalence ranging from 56 to 72% [3–5]. 25% of patients are reported to have severe volume overload [6], demonstrating that current therapies, including hypertonic glucose PD solutions, icodextrin and automated PD (APD), are often insufficient to achieve euvolemia. Importantly, volume overload is associated with increased mortality, morbidity and technique failure [7–9]. Accordingly, novel innovative therapeutic interventions are needed to improve the management of volume overload in PD patients.

**Fig. 1:**
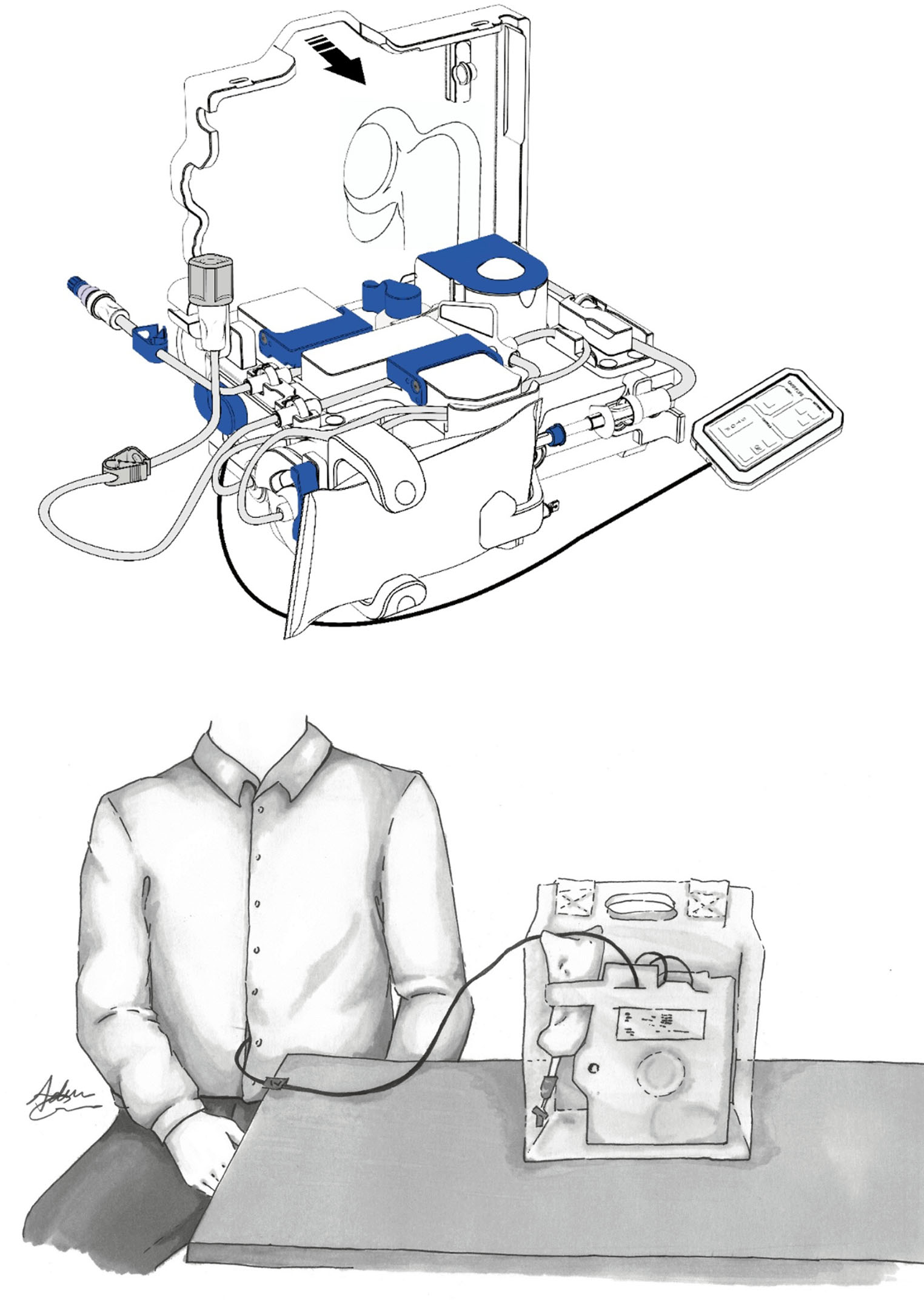
Schematic drawing of the Carry Life UF system (**a**) and a drawing of the device, in the carrying bag, connected to a patient (**b**)

During a PD dwell, glucose diffuses to plasma resulting in a rapidly declining glucose concentration in the dialysate, and hence a loss of the osmotic driving force. The Carry Life UF is a novel system (Fig. 1) using the concept of steady concentration PD (SCPD), where glucose is added continuously during the PD dwell to maintain a stable intraperitoneal glucose concentration, thereby enabling effective UF throughout the duration of the dwell [10]. During the entire treatment, the Carry Life UF device dilutes and mixes a small volume of a concentrated glucose solution with a small portion of the dialysis fluid transferred to the device, before returning it to the patient. The device automatically drains approximately 180 mL of dialysate every hour into a drainage bag [10].

A recent study of SCPD treatments using the Carry Life UF system in the hospital setting resulted in higher UF rates, more efficient use of glucose (increased UF volume/gram of glucose absorbed), and greater sodium removal compared to a control 2.27% glucose continuous ambulatory PD (CAPD) dwell [10]. During this study the Carry Life UF system was used at three different glucose doses (11, 14, and 20 g glucose per hour), for five-hour treatments. The continuous glucose infusion prevented the fall in intraperitoneal glucose concentration that occurs during a standard CAPD dwell and kept the intraperitoneal glucose concentration relatively stable.

The aim of the present study is to compare the efficacy and safety of the Carry Life UF system in adult CAPD patients, using glucose doses of 11 g per hour and 15 g per hour, with a standard CAPD prescription in the home setting.

## Methods/design

**Fig. 2:**
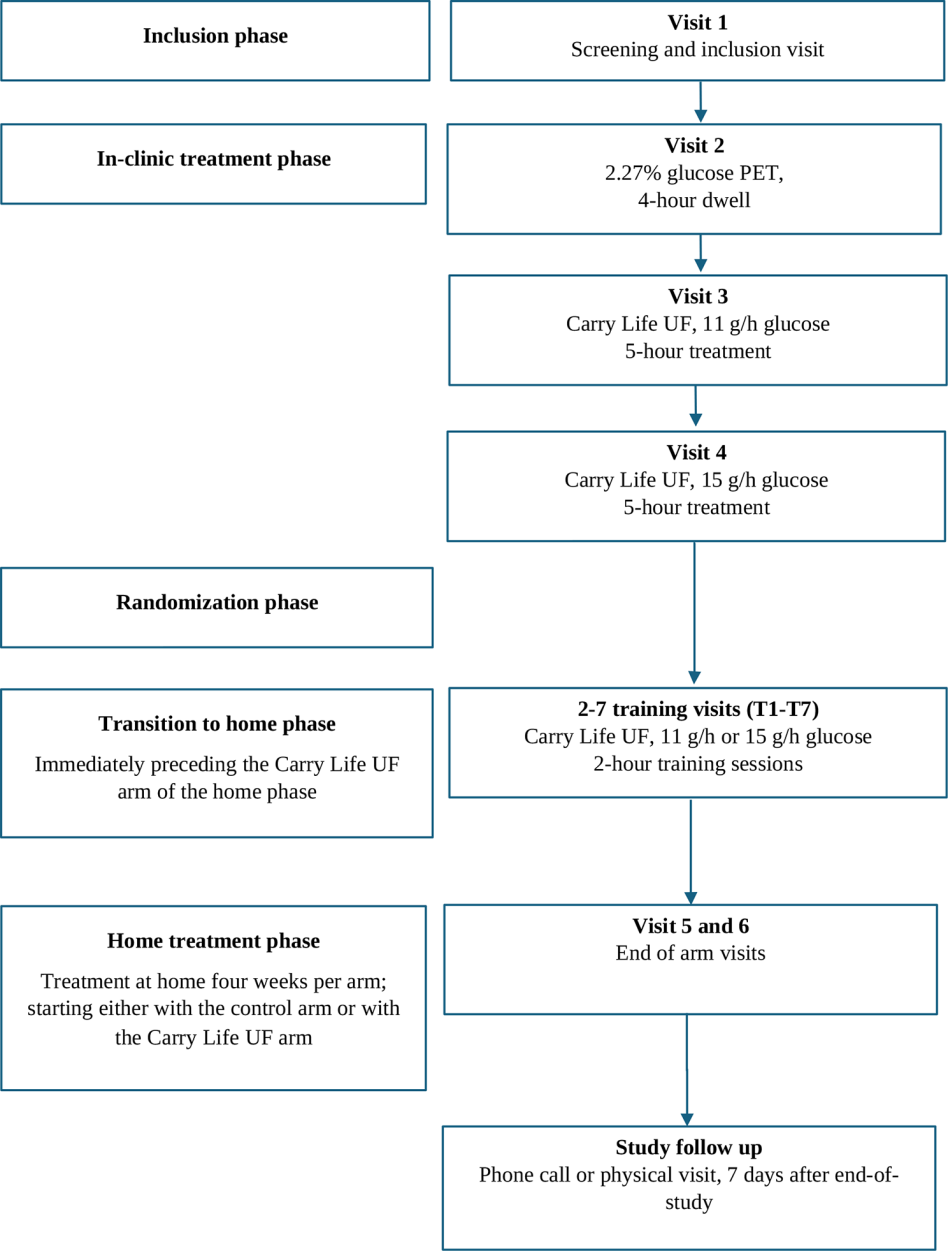
Flow chart over study phases and visits

**Fig. 3:**
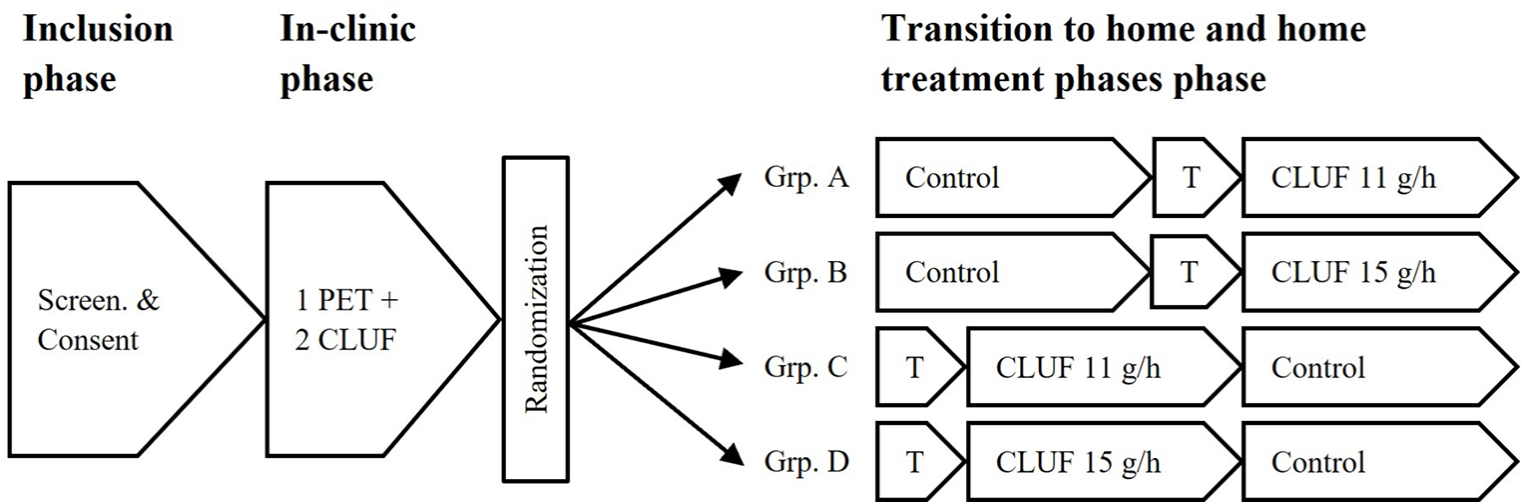
Study flow chart. *Footnote*: CLUF = Carry Life UF; Grp. = Group; PET = Peritoneal equilibration test; Screen. = Screening; T = Transition to home treatment phase with device training

The study consists of five phases (Fig. 2–3), including an in-clinic phase for determination of the Carry Life UF treatment glucose dose (11 g/h or 15 g/h), and a home treatment phase during which the subject performs the control arm and the Carry Life UF arm in random order.

The primary objective of the study is to demonstrate that a 5-hour Carry Life UF treatment at home results in an increased UF volume compared to a 5-hour 2.27% glucose CAPD dwell.

The secondary objectives are:

- To evaluate the overall safety of the Carry Life UF system used by the subject at home as measured by rates of adverse events (AEs) and serious adverse events (SAEs).
- To demonstrate that treatment with the Carry Life UF system at home results in increased sodium removal compared to a 2.27% glucose CAPD dwell.
- To demonstrate that treatment with the Carry Life UF system at home results in a more glucose efficient peritoneal fluid removal compared to a 2.27% glucose CAPD dwell.
- To evaluate if the dialysate peak glucose concentration during a Carry Life UF treatment is lower than the glucose concentration of a 2.27% glucose peritoneal dialysis solution.

The explorative objectives are:

- Evaluation of peritoneal urea and creatinine removal.
- Evaluation of overall weekly UF volumes based on the patient diary.

### Study settings and subjects

Approximately twenty-five adult CAPD patients will be recruited at up to 12 study sites in Italy, Sweden and the UK, subject to study inclusion and exclusion criteria (Table 1), and willingness to sign an informed consent having reviewed the patient information sheet.

**Table 1:** Inclusion and exclusion criteria for the study

**Inclusion criteria**	
Age ≥ 18 years.	
Subjects with end-stage kidney disease (ESKD) treated with PD for at least three months.	
A PD prescription of 2–4 CAPD dwells/day unchanged for a minimum of two weeks, with at least one 1.5–2 L, 2.27% glucose day dwell daily.	
Subjects must be able to tolerate a 2 L PD fill volume for the peritoneal equilibration test (PET).	
Subjects using the Baxter PD system with a MiniCap transfer set.	
In the opinion of the investigator, the subject has the capacity to learn how to use the Carry Life UF system or has a caregiver who can do so.	
Obtained written consent to participate in the study.	
**Exclusion criteria**	
A PD prescription including a regular 3.86% glucose day dwell.	
An episode of peritonitis within the last three months.	
Serum potassium > 6 mmol/L within the last three months.	
Serum urea > 35 mmol/L within the last three months.	
Clinical signs of dehydration.	
Systolic blood pressure < 100 mmHg within the last month.	
Known diagnosis of clinically significant aortic stenosis.	
Clinical condition of unstable diabetes.	
Subjects with a life expectancy of < six months.	
Evidence of any other diseases or medical conditions that may interfere with the planned treatment or affect subject compliance.	
Participation in clinical trials, interfering with the present study, within the previous month.	
Anticipated living donor kidney transplantation within six months of screening.	
Pregnant, breastfeeding, or women of childbearing potential who are not using an effective method of contraception (hormonal contraceptives or barrier contraceptive methods).	

### Compensation to subjects

Due to the significant burden associated with the present trial and to enable a smooth recruitment process, study subjects will be offered a fair compensation for their time and effort, in accordance with local regulations [11].

## Study procedures

An overview of the study activities is presented in Table 2, Supplement 1).

### Inclusion phase

Once the patient has signed the informed consent form and has met the inclusion and exclusion criteria, the subject will be considered eligible to be enrolled in the study.

If the patient does not meet the PD prescription requirement, he/she may consent to participate in the study subject to a change of PD prescription in order to meet the requirement. The change in prescription will be made after assessment by the responsible physician to ensure that the new prescription is clinically comparable to the current prescription. A run-in period of at least two weeks with a PD prescription that meets the inclusion criteria will then be performed.

Patient demographics, medical history, concomitant medications, baseline PD prescription and 24 h UF volume will be recorded. A screening log will be maintained at each study center containing limited non-identifiable information.

### In-clinic treatment phase for dose determination and safety evaluation

The in-clinic phase consists of three visits (visits 2–4). During visit 2, a four-hour peritoneal equilibration test (PET) will be performed for determination of solute transfer rate classification, using a 2 L fill of 2.27% glucose PD solution. During visits 3 and 4, a five-hour Carry Life UF treatment will be performed with the 11 g/h and 15 g/h glucose doses respectively. The Carry Life UF treatments will be used for a safety evaluation, and based on the UF volumes achieved at each treatment, the Carry Life UF glucose dose for the home treatment phase will be determined.

At visit 2, baseline blood chemistry will be collected before treatment. Furthermore, a 24-hour urine collection will be performed in subjects with a urine volume more than 100 mL/24 hours, and urine concentration of creatinine and urea will be measured for calculation of residual kidney function.

At visits 2–4, body weight, systolic and diastolic blood pressure, and heart rate will be measured before and after treatment.

The Carry Life UF treatments start with a manual peritoneal fill of 1.5–2 L, according to the subject’s standard prescription, of a 1.36% glucose PD solution, after which the subject connects to the device.

During the in-clinic treatments (visits 2–4), there will be regular measurements of plasma concentrations of glucose. Refer to section Sampling and analysis for details of the sampling procedures.

If the 11 g/h glucose dose Carry Life UF treatment results in a UF volume of 1.0 L or greater, the 15 g/h glucose dose Carry Life UF treatment will not be performed.

The Carry Life UF glucose dose prescription for the home treatment phase will be determined according to the following criteria:

1. The 15 g/h glucose dose will only be used if both of the following conditions are met:
  The treatment with the 11 g/h glucose dose achieved a UF volume of less than 1.0 L.
  The treatment with the 15 g/h glucose dose achieved a UF volume 25% greater than the treatment with the 11 g/h glucose dose.
2. In all other cases the 11 g/h glucose dose will be used.

Subjects will be withdrawn from the study if during the in-clinic phase they:

1. Experience a systolic blood pressure < 100 mmHg where the hypotension is deemed to be caused by hypovolemia due to excessive peritoneal UF.
2. Generate an UF rate higher than 20 mL/kg body weight/treatment.

Before continuing to the randomization phase, the responsible physician will review the baseline blood chemistry results and the plasma glucose data generated during the in-clinic treatment phase, check that there have been no episodes of systolic blood pressure of < 100 mmHg or UF rate exceeding 20 mL/kg body weight/treatment, and confirm that the subject can proceed to the next phase of the study.

### Randomization

When the glucose dose for the home treatment phase has been determined, subjects will be randomized to start with either the control or the Carry Life UF study arm. For each Carry Life UF glucose dose (11 g/h and 15 g/h), the subjects will be randomized with an equal ratio to start with either the control or the Carry Life UF study arm (Fig. 2). The randomization will be based on block randomization using blocks of four. The randomization will be managed centrally by the electronic case record form (eCRF).

### Transition to home treatment phase

Before starting the Carry Life UF study arm, the study subjects (and their caregivers, in case the subject has a caregiver who normally performs the PD treatment) will undergo daily training on the Carry Life UF device, for 2–5 days, to ensure that the user can operate the device safely and autonomously. The subject/caregiver will be evaluated by the responsible training nurse, and must pass a Carry Life UF system competency assessment before starting the Carry Life UF arm. Each training session and the outcome of the competency assessment will be documented.

If the subject/caregiver does not pass the Carry Life UF system competency assessment, two additional training sessions may be performed, and the competency assessment repeated. If the subject/caregiver still does not pass the competency assessment, the subject will be excluded from the home treatment phase of the study. The number of subjects who do not pass the Carry Life UF system competency assessment will be documented and reported.

### Home treatment phase for efficacy and safety evaluation

Subjects will start in the control or in the Carry Life UF arm according to the randomization. A study nurse will be present for the first Carry Life UF treatment at home to oversee the Carry Life UF system set-up.

In the control arm, subjects will use their regular CAPD prescription. In the Carry Life UF arm, on Mondays, Wednesdays and Fridays, the subject will replace one 2.27% glucose dwell with a Carry Life UF treatment, using the same PD fill volume as in the control arm. On the remaining four days of the week, one 2.27% glucose dwell will be replaced by a 1.36% glucose dwell.

In the Carry Life UF arm, the Carry Life UF system will be incorporated in the subject’s CAPD regimen as follows:

- The preceding CAPD dwell will be ended with a complete peritoneal drain.
- The Carry Life UF treatment starts with a fill of 1.36% glucose PD solution, 1.5–2 L, according to the subject’s standard CAPD prescription.
- After the fill, the Carry Life UF device will be connected to the catheter extension set and the Carry Life UF treatment will be started.
- After five hours the Carry Life UF treatment will end, the device will be disconnected, and the peritoneal fluid will be drained. The subjects will then continue with their standard CAPD prescription.

During the home treatment phase, the subject will record body weight, blood pressure and heart rate daily in a patient diary. Furthermore, the glucose concentration of the PD solutions that are used will be recorded daily, together with the PD fill and drain volumes.

Diabetic subjects will monitor and manage their blood glucose levels in accordance with their normal practice. After initiating Carry Life UF treatments, blood glucose levels measured as part of the subject’s standard diabetes management will be evaluated daily by the study center for at least three days, or until judged clinically stable. Thereafter, blood glucose levels will be evaluated weekly by the center.

At the start of the second and third week of each study arm of the home treatment phase a nurse will contact the subject to check on clinical status, possible AEs, and Carry Life UF device malfunctions. The clinical follow-up will include data on body weight and blood pressure, as well as a clinical assessment of volume status and clinical symptoms.

Based on the clinical assessments, the responsible physician will adjust the subject’s PD prescription in order to maintain an appropriate fluid balance according to clinical judgement and standard clinical practice.

If a reduced peritoneal fluid removal is deemed necessary during the Carry Life UF treatment arm, a change in the prescription shall be performed in the following order.

1. Reduce the glucose concentration of the CAPD dwells.
2. Reduce the frequency of the Carry Life UF treatments.

When a Carry Life UF treatment is removed from the prescription, a 2.27% glucose or 1.36% glucose CAPD dwell will replace the Carry life UF treatment based on the clinical judgement of the responsible physician.

The Carry Life UF treatment should be performed at least twice per week. If the subject requires a Carry Life UF treatment frequency lower than two days per week to avoid hypovolemia, the subject will be withdrawn from the study.

If an increased peritoneal fluid removal is deemed necessary during the Carry Life UF treatment arm, a change in the prescription shall be performed in the following order:

1. Re-introduce the 2.27% glucose CAPD dwells that were replaced by 1.36% glucose dwells (one at the time at a frequency indicated by the subject’s clinical status).
2. When all 2.27% glucose dwells have been re-introduced, one additional Carry Life UF treatment may be added weekly until the fluid removal is deemed sufficient. The added Carry Life UF treatment will always replace a 2.27% glucose CAPD dwell.

In the control arm, the glucose concentration of the PD dwells may be adjusted as required.

All changes to the subject´s PD prescription will be documented and the reason for the change specified.

At four efficacy evaluations days during the home treatment phase (two per each study arm), there will be a careful recording by a study nurse of the solution volumes used (PD solution and glucose solution) and volumes drained, for the calculation of UF volume (primary endpoint). Furthermore, dialysate samples from the control 2.27% glucose 5-hour CAPD dwell and from the Carry Life UF treatment will be collected by a study nurse for secondary endpoint evaluation (peritoneal sodium removal, glucose UF efficacy, peak dialysate glucose concentration). The efficacy evaluation days will be performed during week 2 and week 4 of each study arm, on the same day of the week and at the same time of the day.

On the final day of each study arm the subject will visit the clinic for clinical evaluation, blood chemistry analysis, and measurement of body weight, systolic and diastolic blood pressure and heart rate. Starting two days before the end-of-arm visits, a 24-hour urine collection will be performed in subjects with a urine volume more than 100 ml/24 hours, and urine concentration of creatinine and urea will be measured for calculation of residual kidney function.

### Safety reporting during the study

AEs, SAEs, and Carry Life UF device malfunctions will be recorded throughout the study. A nurse at each participating study center who has undergone education and training necessary for thorough AE collection will be responsible for the recording of AEs, i.e., education in good clinical practice (GCP) will be required and training with respect to the eCRF for AE reporting.

The subject will be instructed to record AEs and Carry Life UF device malfunctions on forms contained in the patient diary. In case of an AE or a device malfunction during the home treatment phase, the subject will be instructed to contact the study center immediately. The contact details of the center will be provided. Furthermore, the center will contact the subject weekly to enquire about the occurrence of any AE or Carry Life UF device malfunction.

The subject should contact the clinic immediately if they have any questions or concerns about their clinical status or about the study in general. If there are any concerns about the subject’s clinical status that cannot be resolved by phone, the subject will be brought to the clinic for a clinical assessment.

Any changes in medication or PD prescription will be recorded during the study. If changes to diuretics or PD prescription are made, the reason for the change will be documented.

If a study subject discontinues the clinical study the reason for this will be documented in detail.

### Sampling and analysis

At visit 2 (the PET) as well as at the visits at the end of each home treatment arm, plasma samples will be collected for analysis of sodium, potassium, magnesium, ionized calcium, phosphate, albumin, creatinine, urea, and parathyroid hormone. At the same visits, 24-hour urine collections will be analyzed for creatinine and urea to determine the residual kidney function.

During the three in-clinic treatments (the PET and the two Carry Life UF treatments), plasma samples will be taken for analysis of glucose concentrations before the treatments, at 30 min, at 1 h and then hourly until the end of treatment.

Dialysate samples during the PET will be collected at 0, 1, 2 and 4 h for analysis of glucose and creatinine for determination of solute transfer rate classification. For sampling, a separate drain bag will be connected after the completion of the PD fill (T0) and at 1 and 2 h, and 200 ml fluid will be drained into the bag, out of which 20 ml will be withdrawn for analysis and the remaining fluid returned into the peritoneal cavity. The 4-hour sample will be taken from the final drain. Sodium concentration at each time points will also be analyzed.

Dialysate samples taken during the in-clinic Carry Life UF treatments will be collected from the Carry Life UF drain bag at 0, 1, 2, 3, 4 and 5 h for analysis of glucose and sodium (the PD fluid is automatically drained by the Carry Life UF system at these timepoints). After sampling, the remaining drained PD fluid will be pooled. The pooled PD fluid, plus the final peritoneal drain will be analyzed for glucose, sodium, potassium, calcium, phosphate, albumin, creatinine, and urea, for calculation of glucose absorption, glucose UF efficiency, and peritoneal removal of the above-mentioned substances.

During the home treatment phase efficacy evaluation treatments, dialysate samples will be collected from the final drain bag and the Carry Life UF drain bag (for Carry Life UF arm only) for analysis of glucose, sodium, potassium, calcium, phosphate, albumin, creatinine, and urea, for calculation of glucose absorption, glucose UF efficiency, and peritoneal removal of the above-mentioned substances.

Analysis of plasma and urine samples will be performed at the local hospital laboratory in association with the visits. Dialysate samples will be stored at a temperature below − 20 °C at each study center and will be transported on dry ice to a central laboratory for analysis.

### Bag weighing procedure

In order to obtain a correct value of the UF volumes during the study consistent across all study centers and to account for PD bag overfill and material weights, detailed instructions of the bag weighing procedure will be provided. The UF volume from a CAPD dwell or Carry Life UF treatment is calculated by subtracting the fill volume (PD solution and glucose solution) from the drain volume as detailed below:

- To determine the PD solution fill volume, the CAPD Y-system fill bag is weighed after the flush and the bag plastic weight is subtracted.
- To determine the glucose fill volume, the weight of the used glucose bag (g) is subtracted from the weight of full glucose bag (g) and the difference is divided by the density of the 50% glucose solution (1.2235 g/mL).
- To determine the drain volume, the CAPD Y-system drain bag is weighed before the flush-before-fill of the new PD solution, and the bag plastic weight is subtracted.

To ensure data quality, UF volumes are automatically calculated in the eCRF. To reduce the impact of measurement variability, the plastic weight of each different type of bag used in the study is assumed to be constant, and the predetermined bag plastic weights are used in the UF calculation algorithms.

### Data collection and Documentation

Data will be collected as described in the above sections and recorded using an eCRF developed for the study. All personal information will be kept at the clinics and only blinded data will be stored in the eCRF.

A patient diary will be filled out during the home treatment phase of the study. For subjects able to use an electronic diary this will be used. Remaining subjects will use a paper diary, and data will be transferred to the eCRF by study staff at the center. The diary includes daily registration of body weight, blood pressure, and heart rate. Furthermore, type of PD fluid, glucose strength, and drain weights of all PD exchanges during the home treatment phase will be registered. If the subject experiences any problems during the home treatment phase there will be an option to register free text in the diary.

Due to size of the study and the limited additional risk from treatments a data monitoring committee is not needed.

### Monitoring

Qualified monitors from an independent clinical research organization will conduct the monitoring activities according to a specific plan including regular monitoring visits during the clinical investigation, and at close-out. 100% source data verification will be performed with exception of the patient diary. The monitoring activities will be conducted according to GCP.

### Sample size calculation

The primary objective of the study is to demonstrate that a 5-hour Carry Life UF treatment at home results in an increased UF volume compared to a 5-hour 2.27% glucose CAPD dwell. A UF volume ≥ 250 mL greater with the Carry Life UF treatment than with the 2.27% glucose CAPD dwell is considered a clinically relevant UF volume.

The sample size is calculated using the UF volume data from a previous pilot study [10] of eight subjects, with the aim to demonstrate a superiority margin, delta (δ), of 250 mL. For each subject the delta (δ) UF volume between the Carry Life UF treatment and the control 2.27% CAPD dwell (paired comparison) at two glucose doses (11 g/h and 14 g/h) provided input to the sample size calculation. The difference between the Carry Life UF and the control 2.27% CAPD dwell had a mean value of 574 mL and a standard deviation of 236 mL.

The sample size was estimated by:

$$\:n=\:\frac{{({z}_{\alpha\:}+{z}_{\beta\:})}^{2}{\sigma\:}_{m}^{2}}{2{(\epsilon-\delta\:)}^{2}}$$

where

ε = test - control.

δ = superiority margin.

σ_m_ = standard deviation of the paired differences.

Set.

Alpha = 0.025, Z_alpha = 1.96.

Power = 0.80, Z_beta = 0.842.

σ_m_ = 236.

ε = 574.

δ = 250.

Assuming a standard deviation of 236 mL, an expected difference of 574 mL and a superiority margin of 250 mL requires a sample size of 3 to obtain 80% power for the superiority test (α = 0.025) using a one sample superiority (one-sided) t-test sample size calculation.

We also consider a more extreme case where we assume a standard deviation of 1.4 times the previous standard deviation, or 330 mL, Further, we lower our assumed mean difference to 400 mL (about 0.7 times below the previous observed value). With these assumptions (a standard deviation of 330 mL, an expected difference of 400 mL and a superiority margin of 250 mL), we require a sample size of 19 to obtain 80% power for the superiority test (α = 0.025).

The study size of 25 was selected based on the conservative sample size with additional subjects to account for possible dropouts.

### Statistical analysis

#### Statistical analysis of primary endpoint

The primary endpoint uses the UF volume measured per protocol at each efficacy evaluation treatment during the home treatment phase. Each subject is expected to have two efficacy evaluations per treatment arm. Subjects with one or more per protocol efficacy evaluation in each arm will be included in the primary endpoint analysis. The analysis will be performed for both the intention-to-treat (ITT) and the per protocol (PP) population (Table 3, Supplement 2). Excluded subjects will be tabulated with narratives as to why they are missed from the efficacy evaluations.

For each subject, the mean UF_CL_ and the mean UF_CAPD_ will be used for analysis. The mean difference (i.e., a paired comparison) between the UF volume achieved with the Carry Life UF treatment and the UF volume achieved with the control CAPD dwell (UF_CL_(n) – UF_CAPD_) will be calculated (UFdiff(n)) with a 95% confidence interval (CI). A superiority (one sided t-test) test will be used to demonstrate that the Carry Life UF treatment is superior to the control treatment with superiority margin of 250 mL. A superiority margin greater than 0 mL but less than 250 mL supports that the Carry Life UF treatment increases the UF volume as compared to the control treatment, but at a level that may not be clinically significant.

For the primary endpoint analysis, the four groups (Group A, 11 g/h glucose dose starting with control; Group B, 15 g/h glucose dose starting with control, Group C, 11 g/h glucose dose starting with Carry Life UF; Group D, 15 g/h glucose dose starting with Carry Life UF) in the randomization scheme (Fig. 2) will be pooled. Control data generated from all four groups will be part of the control group and Carry Life UF data for both glucose doses from all four groups will be part of the Carry Life UF group. This results in two final groups for primary endpoint analysis; control and Carry Life UF. Based on (1) the prior pilot study results showing no significant difference between the doses and (2) the dose determination procedure, which assigns the glucose dose to be used by each subject based on the UF volume result in the in-clinic phase of the study, we expect the two Carry Life UF glucose dose groups to behave as a single population and be poolable for the purposes of the primary endpoint of the study. However, as part of our descriptive analysis, we intend to summarize the difference in UF volume between study arms for each Carry Life UF glucose dose as well as for different fill volumes.

The treatment performance for the primary objective will also be described based on demographic characteristics including age, gender, and ethnicity.

#### Statistical analysis of secondary endpoints

For all secondary endpoints, excluded subjects will be tabulated with narratives as to why they are excluded. A subject may be excluded from one or more secondary endpoint, that is, exclusions are made independently for each endpoint.

##### AE and SAE rates

All safety events reported during the clinical investigation from either arm during the home treatment phase will add information to the safety dataset. Based on all safety data gathered during the study for the ITT dataset, AE and SAE occurrences adjudicated to the Carry Life UF treatment or the control treatment will be collected and summarized. The rates of AE and SAE and the rates of different types of AE and SAE during the Carry Life UF treatment arm and during the control arm will be presented (events per day). Data for the home treatment phase PP data set and the in-clinic treatments will also be gathered and presented. The occurrence of AE and SAE for each Carry Life UF glucose dose will be summarized.

##### Peritoneal sodium removal

For the secondary endpoint peritoneal sodium removal, each subject is expected to have two efficacy evaluation treatments per study arm during the home treatment phase of the study. Subjects with one or more evaluation treatments in each arm will be included in the secondary endpoint analysis. The analysis will be performed for both the ITT and the PP population.

Based on data gathered from the efficacy evaluation treatments, peritoneal sodium removal will be calculated. For each patient the average sodium removal from the per protocol efficacy evaluation treatments in each arm will be used for the analysis. A paired comparison between sodium removal (NaRev) achieved with the Carry Life treatment (NaRev_CL_) and sodium removal from the control CAPD dwell (NaRev_CAPD_) will be conducted. The average paired difference and the associated 95% CI will be calculated. Further, a t-test will be used to demonstrate that the Carry Life UF treatment has a larger peritoneal sodium removal than the control 2.27% CAPD dwell. The difference in peritoneal sodium removal for each Carry Life UF glucose dose and each fill volume compared to the control CAPD dwell will be summarized.

##### Glucose ultrafiltration efficiency

For the secondary endpoint glucose UF efficiency, each subject is expected to have two efficacy evaluation treatments per study arm during the home treatment phase of the study. Subjects with one or more evaluation treatments in each arm will be included in the secondary endpoint analysis. The analysis will be performed for both the ITT and the PP population.

Based on data gathered from the efficacy evaluation treatments, glucose UF efficiency will be calculated. For each patient the average glucose UF efficiency (ml UF / g glucose absorbed) from the per protocol efficacy evaluation treatments in each arm will be used for the analysis. A paired comparison between the glucose UF efficiency (GlucEff) achieved with the Carry Life UF treatment (GlucEff_CL_) and the control CAPD dwell (GlucEff_CAPD_) per protocol treatments will be conducted. The average paired difference and the associated 95% CI will be calculated. Further, a t-test will be used to demonstrate that the Carry Life UF treatment has a higher glucose UF efficiency than the control 2.27% CAPD dwell. The difference in glucose UF efficiency for each Carry Life UF glucose dose and each fill volume compared to the control 2.27% CAPD dwell will be summarized.

##### Peak dialysate glucose concentration

During the in-clinic phase of the study, the peak dialysate glucose concentration during the Carry Life UF treatments will be determined from the hourly dialysate sampling points for each glucose dose. For this secondary endpoint, each subject will have dialysate glucose data from one in-clinic treatment at each Carry Life UF glucose dose.

The average peak glucose dialysate concentration (in %; gram glucose per 100 mL fluid) for each glucose dose of the Carry Life UF in-clinic treatments and the associated 95% CI will be calculated for both the ITT and the PP population. Further, a t-test will be used to demonstrate that the peak dialysate glucose concentration during the Carry Life UF treatment is lower than 2.27%. The peak glucose concentration at each fill volume will be summarized.

#### Statistical analysis of exploratory endpoints

A basic comparison of the peritoneal removal of creatinine and urea as well as of the weekly UF volumes based on the patient diary will be performed across the arms of the study.

#### Description and interference

Aggregated continuous data will be presented in terms of mean, median, standard deviation, minimum and maximum, and number of observations. Aggregated categorical data will be presented using frequency tables.

#### Missing values

Subjects who drop out of the study will be characterized and compared to those remaining in the study based on demographics and co-morbidities.

#### Additional summaries

All demographic and baseline characteristics captured in the eCRF will be summarized for all populations included in the analysis (ITT and PP as defined for all endpoint analysis), and descriptive statistics will be presented when applicable. The frequency and distribution of missing data for any characteristic will also be described.

#### Listings

All data captured in the study will be listed. This will include outcomes not included in the summaries such as concomitant medication and medical history.

## Discussion

The aim of this prospective, multicenter, randomized, crossover study is to assess the efficacy and safety of the Carry Life UF system used at home in adult patients treated with CAPD. It follows on from a recently published in-center study in eight subjects, which showed that SCPD with the Carry Life UF system resulted in higher UF rates, more efficient use of glucose, and greater sodium removal [10]. The main differences compared to the previous study are that more subjects will be included (19 subjects will complete the investigation), more Carry Life UF treatments will be performed per subject, the Carry Life UF treatments (three per week) will be incorporated into the subject’s standard CAPD prescription for four weeks, and, most importantly, the Carry Life UF treatments will be performed in the home setting. The primary outcome measure is comparative UF, however, the protocol design takes a cautious approach to avoid exposing subjects to excessive cumulative fluid removal. This has been done by replacing one 2.27% glucose CAPD dwell with a 1.36% glucose CAPD dwell during the intervention phase, on the four days of the week when the Carry Life UF system is not being used. This is estimated to result in a similar UF volume and glucose exposure on a weekly basis in the two study arms, while allowing direct comparison of the Carry Life UF treatment with a standard 2.27% glucose CAPD dwell with respect to endpoint analyses.

An important component of the study is to gain experience from end-users of the Carry Life UF system in the home setting. The study will provide information on the number of training sessions and the total hours of training required before allowing the subjects to use the system at home. This training will be delivered by experienced PD nurses who have received specific education on the Carry Life UF system. The four-week period using the Carry Life UF system three times per week will result in 228 treatments at home, giving ample opportunity to evaluate the performance and safety of this novel technology.

Correct determination of UF volumes in PD studies is a challenge. Important considerations, such as bag overfill and weights of plastic material have been reported as often being overlooked and/or insufficiently described in publications, making comparison between studies difficult. For instance, the variability in PD bag volumes can be significant, which may create errors up to 200 mL in a 2 L exchange and may vary between different brands of PD solution [12, 13]. Moreover, the flush-before-fill procedure may vary considerably between clinics as well as among patients. In the present study, the measurement of UF volume has been meticulously planned to ensure a correct analysis. Detailed weighing and sampling instructions will be provided to the study centers to ensure accurate data quality. This includes considering the flush-before-fill volume for the PD fill, as well for the PD drain measurements. Measures to ensure reliable endpoint data include: (1) weighing of the PD fill bag is performed after the flush and before the fill, (2) weighing and sampling of the CAPD Y-set drain bags are performed before the flush-before-fill procedure, (3) plastic material weight are considered in the fluid volume calculations, (4) the study centers are provided detailed weighing instructions, such as how to put the bags on the scale and weighing the bags with or without overwrap, and (5) having specially trained study nurses to visit the subjects in their homes to perform the weighing and sampling to obtain data for the endpoint analyses.

Device trials in the home environment typically cause a considerable burden for subjects with respect to time and effort. In this study subjects are required to spend three days at the clinic during the in-clinic phase. During the home treatment phase, they are restricted to their homes during the 5-hour intervention treatments (12 times during four weeks), and further they are requested to complete a daily PD diary over eight weeks. Due to the significant burden associated with the present trial and to enable a smooth recruitment process, it is necessary to offer study subjects a fair compensation, in accordance with local regulations.

In summary, this study sets out to evaluate a novel PD technology based on SCPD, aimed at improving volume management in PD patients in the home environment. The study is associated with a considerable burden on the subjects, furthermore, the execution of the study in the home environment entails challenges both in ensuring accurate endpoint data and in providing necessary support to the subjects in the use of the technology. The protocol has been carefully designed to attend to these issues and to optimize the possibility of the study to reach its stated goals and enable this novel PD system to be used to the benefit of patients treated with PD.

## Electronic supplementary material

Below is the link to the electronic supplementary material.

Supplementary Material 1

Supplementary Material 2

## Data Availability

No datasets were generated or analysed during the current study.

## Declarations

### Insurances

The sponsor will maintain appropriate clinical study insurance coverage as required under applicable laws and regulations for the duration of the clinical investigation.

## Abbreviations

AE: Adverse Event
APD: Automated Peritoneal Dialysis
CAPD: Continuous Ambulatory Peritoneal Dialysis
CI: Confidence Interval
CLUF: Carry Life UF
D: Dialysate concentration
D0: Dialysate concentration at time = 0
eCRF: electronic Case Record Form
ESKD: End-Stage Kidney Disease
GCP: Good Clinical Practice
GlucEff: Glucose UF Efficiency
Grp: Group
ITT: Intention-To-Treat
NaRev: Sodium Removal
P: Plasma concentration
PD: Peritoneal Dialysis
PET: Peritoneal Equilibration Test
PP: Per Protocol
SAE: Serious Adverse Event
SCPD: Steady Concentration Peritoneal Dialysis
Screen: Screening
T: Transition to home treatment phase with device training
UF: Ultrafiltration
