# Supplementary material for: A clinical study of efficacy and safety of the Carry Life UF system in continuous ambulatory peritoneal dialysis patients: protocol for a prospective, multicenter, randomized, crossover study
Source: BMC Nephrol. 2025 Apr 3;26:174. doi: 10.1186/s12882-025-04095-2 (PMC11969901; doi:10.1186/s12882-025-04095-2)
Supplement: Supplementary file 1 — Supplementary Material 1 [file 12882_2025_4095_MOESM1_ESM.docx]

#### **Table 2** Schedule of study activities

| **Visit** | | **V1**  **Screening & Inclusion** | **V2-V4**  **In-clinic treatments** | **Randomization** | **T1-T7**  **Device training** | **Home treatment (two sampling days)** | **V5** | **T1-T7**  **Device training** | **Home treatment (two sampling days)** | **V6/End of study** |
| --- | --- | --- | --- | --- | --- | --- | --- | --- | --- | --- |
| Time point/duration | Group A, B | -14–0 Days | Week 1–2 | Week 2 |  | Week 3–6 | Week 6 | Week 7 | Week 8–11 | Week 11/After premature ending of study |
|  | Group C, D |  |  |  | Week 3 | Week 4–7 | Week 7 |  |  |  |
| Informed consent | | X |  |  |  |  |  |  |  |  |
| Inclusion and exclusion criteria | | X |  |  |  |  |  |  |  |  |
| Demographics^a^ | | X |  |  |  |  |  |  |  |  |
| Medical history^b^ | | X |  |  |  |  |  |  |  |  |
| Concomitant medications^c^ | | X | X |  | X | X | X | X | X | X |
| Baseline PD prescription^d^ | | X |  |  |  |  |  |  |  |  |
| Fluid restriction^e^ | | X | X |  | X | X | X | X | X | X |
| Randomization | |  |  | X |  |  |  |  |  |  |
| Vital signs & body weight^f^ | |  | X |  |  | X | X |  | X | X |
| Food intake and fluid intake & urinary output^g^ | |  | X |  |  |  |  |  |  |  |
| Blood samples^h^ | |  | X |  |  |  | X |  |  | X |
| Dialysate samples^i^ | |  | X |  |  | X |  |  | X |  |
| PD bags used^j^ | |  | X |  |  | X |  |  | X |  |
| Fill and drain volumes^k^ | |  | X |  |  | X |  |  | X |  |
| 24-hour urine sample^l^ | |  | X (V2 only) |  |  |  | X |  |  | X |
| 24-hour UF volume^m^ | |  |  |  |  | X |  |  | X |  |
| Carry Life UF safety assessment^n^ | |  | X |  |  |  |  |  |  |  |
| Transition to home evaluation | |  | X |  |  |  |  |  |  |  |
| Carry Life UF system competency assessment | |  |  |  | X |  |  | X |  |  |
| Subject clinical evaluation^o^ | |  |  |  |  | X |  |  | X |  |
| AEs and SAEs^p^ | |  | X |  | X | X | X | X | X | X |
| Device malfunctions^q^ | |  | X |  | X | X |  | X | X |  |

**Footnotes:**

1. Year of birth, age, gender, ethnicity, height, body weight.
2. Cause of end-stage kidney disease (ESKD), year of diagnosis of kidney disease, start of peritoneal dialysis (PD), start of ESKD therapy other than PD (if applicable) and comorbidities.
3. Concomitant medications will be recorded. Any changes in prescription of medications will be recorded throughout the study, for diuretics and diabetes medication, the reason for change of prescription will be documented.
4. Baseline PD prescription: volumes and glucose concentration for the day dwells, volume for the icodextrin, and volume and glucose concentration for glucose‑based night dwell, as applicable.
5. Any changes in fluid restriction will be recorded throughout the study. The reason for change in fluid restriction will be documented.
6. **In-clinic phase**: Body weight, systolic and diastolic blood pressure, and heart rate will be measured before and after treatment.

**Home phase**: The subjects will record body weight, systolic and diastolic blood pressure, and heart rate daily.

1. **In-clinic phase**: Food intake and fluid intake as well as urinary output during the in-clinic visits will be documented.
2. **In-clinic phase and home phase**: The following plasma (P) chemistry parameters will be analyzed at visit 2 (baseline), visit 5, and visit 6: Sodium, potassium, magnesium, ionized calcium, phosphate, albumin, creatinine, urea, and parathyroid hormone. Creatinine and urea at visits 2, 5, and 6 will be used for calculation of residual kidney function. Plasma creatinine before the peritoneal equilibration test (PET) will be used for calculation of D/P creatinine.

In addition, plasma glucose will be measured before the treatment, at 30 min, at 1 h and then hourly until the end of treatment for the three in-clinic treatments.

1. **In clinic phase**: PET: Dialysate (D) samples will be collected at 0, 1, 2 and 4 hours for analysis of dialysate glucose and creatinine for determination of solute transfer rate classification (4 h D/P creatinine and D/D0 glucose ratios). Sodium concentration at each time point will also be analyzed. After the completion of infusion (T0) and at 1 and 2 hours, 200 mL of dialysate will be drained. A 20 mL sample will be taken, and the remaining 180 mL will be infused back into the peritoneal cavity.

Carry Life UF treatments: Dialysate samples at 0, 1, 2, 3, 4 and 5 hours will be analyzed for dialysate glucose and sodium for evaluation of dialysate glucose and sodium concentrations during treatment. All PD fluid automatically drained (and any additional drains) during the Carry Life UF treatment will be pooled. The pooled PD fluid, plus the final peritoneal drain will be analyzed for dialysate glucose, sodium, potassium, calcium, phosphate, albumin, creatinine, and urea for calculation of glucose absorption, glucose ultrafiltration (UF) efficiency, and peritoneal removal of the listed substances.

**Home phase efficacy evaluation days, during week 2 and week 4 or each arm:** Drained dialysate from the control and from the Carry Life UF efficacy evaluation treatments (Carry Life UF drain bag + final peritoneal drain) will be collected by a study nurse for analysis of dialysate glucose, sodium, potassium, calcium, phosphate, albumin, creatinine, and urea, for calculation of glucose absorption, glucose UF efficiency, and peritoneal removal of the listed substances.

1. **In-clinic phase**: The volume and glucose concentration of PD bags used for the PET and Carry Life UF treatments will be recorded.

**Home treatment phase:** The subjects will record information of all PD bags used (volume and glucose concentration for glucose-based solutions and volume for icodextrin).

1. **In-clinic phase:** The PD bag and glucose bag will be weighed by a clinical professional before the treatment and the drain bag(s) will be weighed after the treatments for calculations of fill volume, drained volume and UF volume.

**Home treatment phase:** The subject will record fill and drain weights of all dwells every day during the home treatment phase.

**Home treatment phase efficacy evaluation days**: A study nurse will weigh the solution bags (PD bag and glucose bag), and the drain bags for calculations of fill volume, drained volume and UF volume.

1. 24-hour urine volume and urine concentration of creatinine and urea will be collected for calculation of residual kidney function.
2. The patient will weigh the PD bag, glucose bag and drain bag(s) for each CAPD dwell/Carry Life UF treatment during the home treatment phase of the study for evaluation of weekly UF volume.
3. To assess the Carry Life UF safety at the different glucose doses, each in-clinic Carry Life UF treatment will be evaluated for the following:

1) a systolic blood pressure of < 100 mmHg.

2) a UF rate higher than 20 mL/kg body weight/treatment.

1. Subjects will be contacted weekly for evaluation of body weight, blood pressure, volume status and clinical symptoms.
2. A nurse adequately trained in adverse event (AE) reporting will specifically ask the subject for occurrences of AEs and serious AEs (SAEs) during a weekly call.
3. The nurse will specifically ask the subject for occurrences of Carry Life UF device malfunctions weekly.
