# Supplementary material for: A clinical study of efficacy and safety of the Carry Life UF system in continuous ambulatory peritoneal dialysis patients: protocol for a prospective, multicenter, randomized, crossover study
Source: BMC Nephrol. 2025 Apr 3;26:174. doi: 10.1186/s12882-025-04095-2 (PMC11969901; doi:10.1186/s12882-025-04095-2)
Supplement: Supplementary file 2 — Supplementary Material 2 [file 12882_2025_4095_MOESM2_ESM.docx]

#### **Table 3** Definition of intention6 54 -to-treat and per protocol populations per different endpoints

| Endpoint Description | Intent-to-treat (ITT) population | Per protocol (PP) population |
| --- | --- | --- |
| Primary Endpoint | | |
| Primary endpoint (UF volume) | Subjects with data from one or more per protocol efficacy evaluation treatments in each study arm. | Subjects with two per protocol efficacy evaluation treatments performed on the planned efficacy evaluation day and dwell of the day in each study arm. |
| Secondary Endpoints | | |
| Safety endpoint (AEs and SAEs) | All the included subjects. | All subjects who complete four weeks’ treatments in each study arm during the home treatment phase. |
| Peritoneal sodium removal and  Glucose UF efficiency endpoints | Subjects with data from one or more per protocol efficacy evaluation treatments in each study arm. | Subjects with two per protocol efficacy evaluation treatments performed on the planned efficacy evaluation day and dwell of the day in each study arm. |
| Peak dialysate glucose concentration | Subjects with in-clinic Carry Life UF per protocol treatments with at least one glucose dialysate result between T1h and T5h per glucose dose. | Subjects with in-clinic Carry Life UF treatments dialysate glucose samples from at least four time points between T1h to T5h. |
